# The R0 package: a toolbox to estimate reproduction numbers for epidemic outbreaks

**DOI:** 10.1186/1472-6947-12-147

**Published:** 2012-12-18

**Authors:** Thomas Obadia, Romana Haneef, Pierre-Yves Boëlle

**Affiliations:** 1 INSERM, U707, Paris, France; 2 APHP, Saint-Antoine Hospital, Paris, France; 3 UPMC Univ Paris 06, UMR-S, 707, Paris, France; 4 EHESP, Paris, France

**Keywords:** Epidemics, Software, Reproduction number, Estimation, R package

## Abstract

**Background:**

Several generic methods have been proposed to estimate transmission parameters during an outbreak, especially the reproduction number. However, as of today, no dedicated software exists that implements these methods and allow comparisons.

**Results:**

A review of generic methods used to estimate transmissibility parameters during outbreaks was carried out. Most methods used the epidemic curve and the generation time distribution. Two categories of methods were available: those estimating the initial reproduction number, and those estimating a time dependent reproduction number. We implemented five methods as an R library, developed sensitivity analysis tools for each method and provided numerical illustrations of their use. A comparison of the performance of the different methods on simulated datasets is reported.

**Conclusions:**

This software package allows a standardized and extensible approach to the estimation of the reproduction number and generation interval distribution from epidemic curves.

## Background

In 2009, the new influenza virus A/H1N1 rapidly spread worldwide [1]. In the World Health Organization guidance document [2] detailing the epidemiological parameters to quickly determine after identification of the disease were: the incubation period, i.e. time between infection and symptoms; the serial interval, i.e. time between symptoms onset in primary and secondary cases; and the initial reproduction ratio, i.e. the average number of secondary cases per primary case. In a systematic review of all articles presenting such estimates for the 2009 H1N1 influenza pandemic [3], we found high variability in the methods used to estimate the same parameters.

Numerical differences in the reported estimates were therefore due in part to the chosen method. Applying all methods on the same dataset would help to understand what variation is due to the method, and this will be encouraged if the required code is widely distributed. It is also worth noting that subtile variation arises from the actual implementation of the same methods. For example, the initial “exponential growth rate” of the epidemic curve, used in the method described by Wallinga & Lipsitch [4] has been estimated using linear regression on logged incidence [5], Poisson regression on incidence data [6] or renewal equations [7].

Authors may have provided code implementing their methods, but no effort has yet been made to provide end users with a unique framework, with standardized approach allowing easy comparisons. To allow comparisons and provide more standardized approaches, we developed an R package implementing five methods that were the most commonly used during the 2009 H1N1 influenza pandemic. These methods are “plug-in” methods, requiring only data that are commonly recorded during an outbreak (epidemic curve, serial interval), and have been applied in a variety of situations.

After briefly recalling the principle of these methods, we illustrate their use, propose some tools to critically examine results and finally discuss applicability and limitations.

### Implementation

We recall and describe the implementation of methods to estimate the serial interval distribution and reproduction numbers in epidemics. We also propose tools to explore the sensitivity of estimates to required assumptions.

### Defining a generation time distribution

The generation time is the time lag between infection in a primary case and a secondary case. The generation time distribution should be obtained from the time lag between all infectee/infector pairs [8]. As it cannot be observed directly, it is often substituted with the serial interval distribution that measures time between symptoms onset. In our software package, the ‘generation.time’ function is used to represent a discretized generation time distribution. Discretization is carried out on the grid [0,0.5), [0.5, 1.5), [1.5, 2.5), etc.… where the unit is a user chosen time interval (hour, day, week…). Several descriptions are supported: “empirical” requiring the full specification of the distribution, or parametric distributions taken among “gamma”, “lognormal” or “weibull”. In the latter case, the mean and standard deviation must be provided in the desired time units.

A function (‘est.GT’) is also provided to estimate the serial interval distribution from a sample of observed time intervals between symptom onsets in primary cases and secondary cases by maximum likelihood.

### Estimation of initial reproduction numbers

Reproduction numbers may be estimated at different times during an epidemic. In the following, we recall methods for estimating the “initial” reproduction number, i.e. at the beginning of an outbreak, and for estimating the “time-dependent” reproduction number at any time during an outbreak, as well as the required hypotheses for the methods. Proposed extensions and options implemented in the software are also presented.

### Attack rate (AR)

In the classical SIR model of disease transmission, the attack rate (AR : the percentage of the population eventually infected) is linked to the basic reproduction number [9], by $R_{0}\;=\;-\frac{\log\left(\frac{1-\text{AR}}{S_{0}}\right)}{\mathrm{AR}-\left(1-S_{0}\right)}$ where *S*_0_ is the initial percentage of susceptible population. The required assumptions are homogeneous mixing, closed population, and no intervention during the outbreak.

### Exponential growth (EG)

As summarized by Wallinga & Lipsitch [4], the exponential growth rate during the early phase of an outbreak can be linked to the initial reproduction ratio. The exponential growth rate, denoted by r, is defined by the per capita change in number of new cases per unit of time. As incidence data are integer valued, Poisson regression is indicated to estimate this parameter [6,10], rather than linear regression of the logged incidence. The reproduction number is computed as $R\;=\;\frac{1}{M\left(-r\right)}$ where M is the moment generating function of the (discretized) generation time distribution. It is necessary to choose a period in the epidemic curve over which growth is exponential. We propose to use the deviance based R-squared statistic to guide this choice. No assumption is made on mixing in the population.

### Maximum likelihood estimation (ML)

This model, proposed by White & Pagano [11], relies on the assumption that the number of secondary cases caused by an index case is Poisson distributed with expected value R. Given observation of (*N*_0,_*N*_1_, …, *N*_*T*_) incident cases over consecutive time units, and a generation time distribution w, R is estimated by maximizing the log-likelihood $\text{LL}\left(R\right)\;=\;\sum_{t=1}^{T}\text{log}\left(\frac{e^{-\mu_{t}}{\mu_{t}}^{N_{t}}}{N_{t}!}\right)$ where *μ*_*t*_  =  *R*  ∑ _*i* = 1_^*t*^*N*_*t* − *i*_*w*_*i*_. Here again, the likelihood must be calculated on a period of exponential growth, and the deviance R-squared measure may be used to select the best period. No assumption is made on mixing in the population.

The approach assumes that the epidemic curve is analysed from the first case on. If this is not the case, the initial reproduction number will be overestimated, as secondary cases will be assigned to too few index cases: we implemented a correction as described in Additional file 1: Supplementary material S1. It is also possible to account for importation of cases during the course of the epidemic.

### Sequential bayesian method (SB)

This method, although introduced as “real-time bayesian” by its authors, more exactly allows sequential estimation of the initial reproduction number. It relies on an approximation to the SIR model, whereby incidence at time *t* + 1, *N*(*t* + 1) is approximately Poisson distributed with mean *N*(*t*)*e*^(*γ*(*R* − 1))^[12], where $\frac{1}{\gamma }$ the average duration of the infectious period. The proposed algorithm, described in a Bayesian framework, starts with a non-informative prior on the distribution of the reproduction number R. The distribution is updated as new data is observed, using $P\left(R|N_{0},\ldots ,N_{t+1}\right)=\frac{P\left(N_{t+1}|R,N_{0},\ldots ,N_{t}\right)\;P\left(R|N_{0},\ldots ,N_{t}\right)}{P\left(N_{0},\ldots ,N_{t+1}\right)}$. In other words, the prior distribution for R used on each new day is the posterior distribution from the previous day. At each time, the mode of the posterior may be computed along with the highest probability density interval. As before, the method requires that the epidemic is in a period of exponential growth, i.e. it does not account for susceptible depletion; it implicitly uses an exponential distribution for the generation time; and assumes random mixing in the population.

### Estimation of time dependent reproduction numbers (TD)

The time-dependant method, proposed by Wallinga & Teunis [13], computes reproduction numbers by averaging over all transmission networks compatible with observations. The probability *p*_*ij *_that case i with onset at time ti was infected by case j with onset at time tj is calculated as $p_{ij}\;=\;\frac{N_{i}\;w\left(t_{i}-t_{j}\right)}{\sum_{i\neq k}N_{i}w\left(t_{i}-t_{k}\right)}$. The effective reproduction number for case j is therefore *R*_*j*_  =  ∑_*i*_*p*_*ij*_, and is averaged as $R_{t}\;=\frac{1}{N_{t}}\sum_{\left\{t_{j}=t\right\}}R_{j}$ over all cases with the same date of onset. The confidence interval for Rt can be obtained by simulation. Correction for real time estimation, where not yet observed secondary cases are taken into account is possible [14]. It is possible to account for importation cases during the course of the epidemic.

## Results

In the following, we assume that the incidence data is provided on a daily basis, i.e. that the time unit is the day. To use the maximum likelihood and time dependent method, it will be necessary that the generation time distribution is discretized using the same time unit. The user may provide incidence data in the following formats:

- Vector of dates of onset. A list of dates in character or date format is required. An epicurve object from the Epitools package [15] (epitools::epicurve) may also be supplied.

- Vector of incidence counts. In this case, the initial date and/or time step can be supplied separately.

We now illustrate the use of the package using an example dataset from the 1918 influenza pandemic [16], then use simulation to compare methods. The code used for this analysis is given in the Appendix.

### Estimating reproduction numbers

The ‘estimate.R’ function applies the methods described above to a given epidemic curve. Several methods may be used at the same time by listing them in the “methods” argument. In the session code presented in the appendix, a generation time distribution typical of influenza is defined, using a Gamma distribution with mean 2.6 days and standard deviation 1 day [17]. An example dataset (Germany.1918) is loaded from the package.

**Figure 1:**
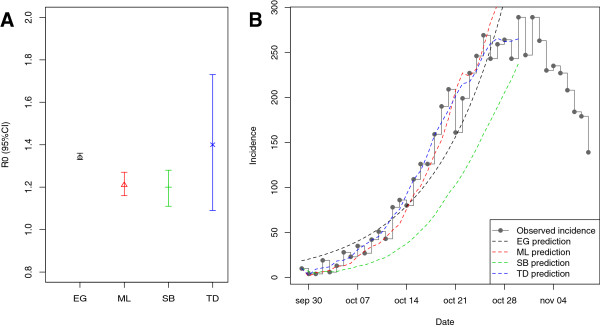
**Estimates of the reproduction ratio and goodness of fit. A**) Estimates of the reproduction ratio by four different methods (see text for details). **B**) Observed incidence (step function) and model predicted incidence for each method.

Initial inspection of the incidence data shows that the exponential growth period takes place during the first 30 days of the epidemic curve. Sensitivity analyses may help refine the choice of an optimal time window for exponential growth (see below). Here, we applied all methods (except the attack rate) on the first 32 days (epidemic peak) of the epidemic curve and reported estimates in Table 1. Surprisingly, although the analysis uses the same data, the estimates range in a relatively large interval, with up to 15% variation (from 1.2 to 1.4). Moreover, confidence or credible intervals do not always overlap (Figure 1A). The fit of each model to the data is however quite similar in all cases, except for the SB method which fits very poorly (Figure 1B).

**Table 1:** Estimation of initial reproduction number by four different methods over the same dataset

**Method**	**Default**	**Optimal**
	**R**	**R**
	**[ 95% CI ]**	**[ 95% CI ]**
EG	1.34	1.56
(optimal time window: 7:22)	[ 1.33 ; 1.36 ]	[ 1.50 ; 1.62 ]
ML	1.21	1.54
(optimal time window: 11:22)	[ 1.16 ; 1.27 ]	[ 1.42 ; 1.66 ]
SB	1.20	1.38
	[ 1.11 ; 1.28 ]	[ 1.25 ; 1.51 ]
TD	1.40	1.40
	[ 1.09 ; 1.73 ]	[ 1.09 ; 1.73 ]

See text for details regarding the methods. All estimates were obtained using the first 32 days of data (default column) or the best fitting time window (“optimal” column). For the SB method, the optimal reported estimate was obtained on day 22, as this date best fits the end of the exponential growth period. For the TD method, daily estimates were averaged over the 32 first days.

### Sensitivity analysis

**Figure 2:**
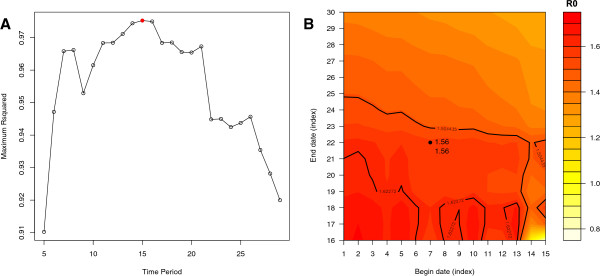
**Sensitivity of the reproduction ratio to the choice of the time period for estimation. A**) Maximum deviance R-squared statistic for time periods of increasing duration. The red dot corresponds with the best value. **B**) Estimates of the reproduction ratio according to various begin and end dates for the time period. The value corresponding to the best fit is shown as a dot, and the solid black lines show the limits of the corresponding 95%CI. In other words, an estimate that falls within the 95%CI of the value showing the best fit can be achieved by using a wide range of begin and end dates. These dates are the ones producing values between the solid black lines.

The EG and ML methods require the user to select the time period over which growth is exponential. By default this is taken as the time period from first case to the date of maximum incidence. However, a better choice is possible using the deviance R-squared statistic over a range of possible time periods. The largest R-squared value corresponds to the period over which the model of analysis fitted the data best: we select this period to provide estimates. To look for this time period, the function ‘sensitivity.analysis’ systematically computes the deviance R-squared statistic over a range of time periods chosen by the user. A plot can be obtained that displays the largest R-squared value over time periods of increasing length (see Figure 2A), and the corresponding estimates can be displayed according to the chosen time window (Figure 2B). Here, this analysis shows that the portion of the epidemic curve that best fitted exponential growth in the EG method was of length 15, and more precisely in this case between time units 7 and 22. The estimate of the reproduction number was 1.56 [ 1.50 ; 1.62 ]. For a large choice of time windows, the estimates of the reproduction number remained within the 95%CI of the best fit, suggesting that the estimate was robust to change in the period of exponential growth. The estimates obtained using the best fitting time window for methods are reported in Table 1. Interestingly, when the “best fitting” time period was used for the EG and ML method, the variability between estimates was reduced.

**Figure 3:**
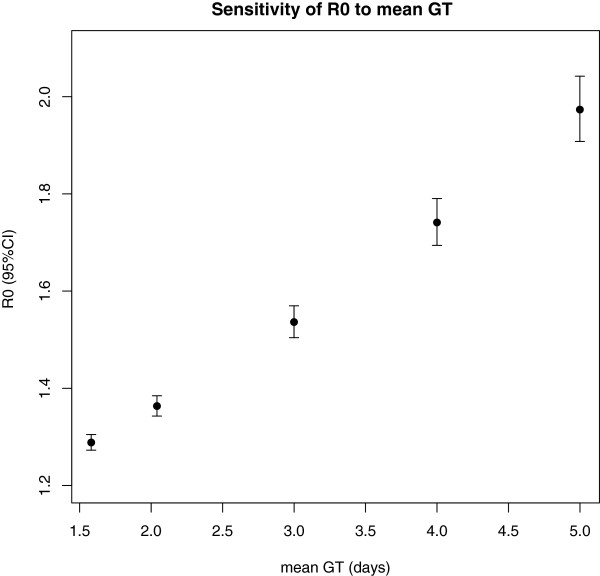
**Sensitivity of the reproduction number to the choice of the generation time distribution.** Reproduction ratio estimates were computed using different mean generation times. Confidence intervals are shown as vertical bars.

A second issue worth considering is how estimates change according to the choice of the generation time distribution. A function was developed that systematically computes the reproduction ratio over a range of user chosen generation time distribution. In our example, we varied the generation time distribution for the EG method (see Figure 3): as expected, the estimates increased with the mean generation time [18]. Using the same epidemic curve, the reported reproduction ratio ranged between 1.3 and 2 when the mean generation time goes between 1.5 and 5 days.

### Comparison of methods

We conducted a simulation study to investigate how estimates of the reproduction number changed with the method of estimation, the role of over-dispersion in the secondary cases distribution and of epidemic curve aggregation at increasingly large time steps. Epidemics were simulated using a branching process, with no restriction on the number of susceptibles to allow exponential growth. For each case, the latent and infectious period were sampled in distributions typical of influenza (gamma distributions with mean+/−sd 1.6+/−0.3 days for latency and 1+/−1 days for infectious period [19]) yielding a generation time interval with mean 2.6 days. The epidemic was started with one incident case at time t=0, then for each incident case, the number of secondary cases was sampled from a negative binomial distribution with mean β I and variance k β I, where I was the individual’s duration of infectious period, β the effective contact rate, and k the overdispersion parameter. The actual time of infection of secondary cases was sampled uniformly during the infectious period of the index case. In this model, the reproduction number is R = β E(I), so that it was possible to calibrate β to obtain the desired value of R. We ran simulations with 3 values for R (1.5, 2 and 3) and overdispersion parameter k to 1 (no overdispersion) and 4 (large overdispersion).

All epidemics were simulated over a period corresponding to approximately the 6 first generations of cases (24 days). For each combination of R and k, 4000 epidemics with more than 20 cases were simulated. Epidemic data were then aggregated daily, by 3 days periods and by 6 days periods. Estimation of the reproduction ratio was made, and the bias and mean squared error were calculated.

The comparison of methods is presented in Table 2. In all cases, when the data were available as daily counts, all methods had approximately the same characteristics. The ML and TD methods were the least biased. Bias increased with larger aggregation periods, especially for the ML and TD methods. For these two methods, the reproduction ratio was increasingly underestimated as data were aggregated on longer periods. Overall, over-dispersion did not significantly affect the estimated value of R. In all cases, the exponential growth method performance was the least affected by either aggregation or over dispersion.

**Table 2:** Bias and MSE of initial reproduction number estimation methods

		**Bias (MSE)**			
**R0**	**Aggregation (days)**	**Method**			
		**EG**	**ML**	**TD**	**SB**
**No overdispersion (k = 1)**					
1.5	1	0.12 (0.0467)	0.02 (0.0164)	0.04 (0.0336)	−0.15 (0.0745)
	3	0.07 (0.0386)	−0.38 (0.1429)	−0.4 (0.16)	−0.1 (0.0277)
	6	0.07 (0.0431)	−0.49 (0.2408)	−0.79 (0.6281)	−0.09 (0.0371)
2	1	0.11 (0.0589)	−0.17 (0.0571)	−0.11 (0.0736)	−0.4 (0.1814)
	3	0 (0.0496)	−0.84 (0.7041)	−0.87 (0.7573)	−0.32 (0.1222)
	6	−0.03 (0.0618)	−0.99 (0.9789)	−1.33 (1.781)	−0.31 (0.1306)
3	1	−0.07 (0.1449)	−0.67 (0.5547)	−0.47 (0.317)	−1.1 (1.2532)
	3	−0.3 (0.2492)	−1.8 (3.2396)	−1.86 (3.4432)	−0.92 (0.8585)
	6	−0.33 (0.2872)	−1.99 (3.9521)	−2.45 (5.9935)	−0.89 (0.8277)
**Large overdispersion (k = 4)**					
1.5	1	0.28 (0.1609)	0.16 (0.0679)	0.33 (0.1913)	−0.05 (0.0881)
	3	0.11 (0.0715)	−0.38 (0.1444)	−0.42 (0.1816)	−0.06 (0.035)
	6	0.08 (0.0694)	−0.49 (0.2415)	−0.83 (0.6906)	−0.04 (0.0574)
2	1	0.13 (0.1109)	−0.15 (0.0696)	0.05 (0.1238)	−0.4 (0.197)
	3	0 (0.086)	−0.84 (0.7108)	−0.89 (0.8007)	−0.32 (0.1328)
	6	−0.03 (0.0988)	−0.99 (0.9792)	−1.36 (1.8686)	−0.3 (0.1517)
3	1	−0.07 (0.2157)	−0.65 (0.5699)	−0.45 (0.3457)	−1.11 (1.2745)
	3	−0.3 (0.3035)	−1.8 (3.2337)	−1.86 (3.4698)	−0.93 (0.898)
	6	−0.36 (0.366)	−1.99 (3.9527)	−2.43 (5.9218)	−0.94 (0.9421)

For each fixed value of R0, 4.000 epidemics were simulated at an individual-based level. A first index case is set at the initial time, and contaminated descendants are sampled in a negative binomial distribution. For each case, we sample a latency period *dlat* during which the individual is infected, but not contagious, and an infectious period *dinf*, both from Gamma distributions of parameters already described for influenza (gamma distributions with mean+/−sd 1.6+/−0.3 days for latency and 1+/−1 days for infectious period [19]). Incidence data are computed as class of time of infection, defined as *t*_*inf*_(*o*) = *t*_*inf*_(*p*) + *dlat*(*p*) + *runif*(1) * *dinf* (*p*), where p is the parent case and 0 the offspring case.

Epidemics that didn’t start due to too few cases were discarded, and estimations were run by batch. For each batch of simulations, we report the bias between the value used to generate the epidemics and the average of all estimates, along with the Mean Square Estimator (MSE) of the simulation series.

## Discussion

We have described a package implementing several methods for estimating the reproduction number from epidemic curves, along with diagnostic tools and provided a comparison of the accuracy of these methods.

The reproduction number in an epidemic is of interest when the disease is actually transmitted between subjects, either directly or indirectly. This is for example the case for influenza, childhood diseases, vector borne diseases, but not in food-borne epidemics caused by environmental exposure to a pathogen. The methods implemented in the package will best be used for acute diseases with short serial intervals. The analysis of diseases with very long incubation times (e.g. HIV or HBV infection) requires more specialized methods, especially to account for censoring [20].

While several methods for estimating reproduction numbers exist, sometimes with code provided by their authors, no common framework was available to allow easy and direct comparison of the results. Developing an R package provide this framework and allow widespread distribution. It will complement package developed for epidemiological surveillance [4] and cost-effectiveness analyses [5]. Furthermore, R packages are easily extensible, so that additional methods can be easily included in future releases.

Regarding the methods described here, we found that when the data was available on a time scale smaller than the mean generation time, all methods tended to be unbiased. Very small aggregation windows may lead to gaps, i.e. time periods with 0 observations. In this case, the SB method fails after the first gap (data not shown). Other methods are not affected, provided the maximum generation time is longer than the gap. When the data was aggregated in time periods up to twice the mean generation time, only the exponential growth method remained unbiased. Indeed, it has previously been reported that aggregation in time periods of 1 mean generation time width was ideal for estimation, and corrections proposed for larger time intervals [21]. The results obtained from the methods described here when data are aggregated in time periods larger than the mean generation time should therefore be interpreted with caution, all the more than the exponential growth assumption is unlikely to be met on long time periods.

In a real situation, and especially for an emerging disease, several practical problems must be taken into account in the application of these methods. The attack rate method requires the least information, but is only usable when the epidemic is over, and furthermore requires that no intervention was set up during the whole course of an outbreak. Therefore its use is generally limited to particular settings like schools or army platoons, for example [22]. All other methods require the epidemic curve and the generation time distribution, with the ‘initial’ reproduction ratio as an output. If one assumes that the population was totally susceptible at first, it may also be interpreted as the basic reproduction ratio (R0); a correction will be necessary in the case of a initial partial immunity.

With a truly emerging outbreak, prior knowledge on the generation time distribution may be unavailable. Allowing sensitivity analyses according to the mean generation time, as we described, is therefore important to help quantify uncertainty in this respect. In other cases, estimation of the generation time distribution is relatively straightforward from pairs of infectors/infectees if there is a marked separation between generations; it is more complicated when generations quickly overlap as with influenza [23]. An additional issue is that symptom onset dates may be known only to an interval, requiring specialized methods for estimation [24]. Joint-estimation of the generation time distribution and reproduction number is another possibility [11,14].

A second step is to choose a time period that displays exponential growth. Too long a time period may depart from true exponential growth and bias estimation downwards, while too short a period may lead to large variance in the estimates. We implemented a method to select the optimal period displaying exponential growth based on the deviance R-squared, a commonly used method to measure goodness of fit of model to data. As shown in Figure 2A, the typical profile of the R-squared deviance presents a maximum that allows selecting the best period.

An implicit assumption in all methods is that all cases are recorded and are linked by a chain of transmission. However, the following issues will arise: missing first cases; under-reporting; unreported cases; reporting delays; importation cases.

If the epidemic is not observed from the first case on, overestimation of the initial reproduction number is likely since some initial cases are absent from the epidemic curve, and secondary cases will be imputed to too few index cases. A correction was implemented for missing generations at the beginning of the epidemic curve for the ML method, similar to that proposed by McBryde in a Bayesian setting [25] using the assumption of constant reproduction number. No obvious way exists to correct the TD method, as the reproduction ratio is allowed to change with time. The EG and SB methods are, by construction, not dependent on this issue.

No method explicitly accounts for under-reporting during the course of the epidemic. If the under-reporting rate is constant in time, no bias is expected. However, if it is known that under-reporting changed with time, this could be corrected before estimation proceeds, as was done in the US [26].

Non-reported cases during an outbreak the epidemic are neither accounted for. Treating missing cases as latent observations has been proposed, but requires the grouping of cases in successive generations rather than on a temporal basis [27].

Importation of cases during the course of the epidemic generally leads to overestimation of the reproduction ratio, since these cases are considered as “offspring” of cases present earlier in the outbreak. In all the methods presented here, only the TD and ML methods can satisfactorily correct for importation cases. The other methods are less easily modified in this respect, and this should be the object of further research.

A final issue is reporting delays, especially when analysis is done in real time. Reporting delays cause a downward bias in incidence in the last few days of observation. This will impact the most the ML and TD estimates, as these rely more heavily on the fit of the model to the observed incidence. In practice, one may wait for data consolidation before applying any method, but it would also be possible to correct for this bias before estimating the reproduction number if the reporting delay is known [14,26].

In the simulation study, we identified that all methods would generally be biased downwards for a disease like flu, with bias increasing both with larger aggregation windows and increasing reproduction number. An exception was the EG method, with upward bias for small R values (R<2) and downwards bias for larger values. When incidence data was available on a daily basis, i.e. smaller than the generation time distribution, the characteristics of the four methods compared. The EG method was the least sensitive to changes in aggregation window, while the ML and TD methods were rapidly inconsistent. The SB method was generally not better than the EG method. Best practice in case of an emerging epidemic will likely depend on a combination of reproduction ratio magnitude, mean generation time duration and aggregation detail. We have provided the framework that would allow comparison and critic of these estimates.

Finally, we highlighted that the estimated reproduction ratio may depend on the method for estimation. This should be taken into account in comparisons, and also when calibrating predictive models as small differences can lead to large variation in attack rates and assessment of required efficacy in interventions.

## Conclusions

Although many mathematical models have been developed to estimate several types of reproduction numbers during epidemic outbreaks, no unique workframe existed. We here provide a user-friendly R package that supports five of the most commonly used methods, and extend some approaches with sensitivity analysis or imputing censored data. This will allow for fast assessment of the transmissibility parameters in new outbreaks, as well as critical assessment of the reported values. The package is currently available from the CRAN repository.

## Availability and requirements

**Project name:** R0 Package

**Project home page:** CRAN repository (http://cran.r-project.org/web/packages/R0/)

**Operating system(s):** Platform independent

**Programming language:** R

**Other requirements:** R v2.13 or higher

**License:** GPL (>= 2)Any restrictions to use by non-academics: None

## Appendix

Typical session code> library(R0) # loads library> # epidemic curve can be input as a list of dates> epid = c("2012-01-01", "2012-01-02", "2012-01-02", "2012-01-03")> # or as incidence counts> epid.count = c(1,2,4,8)> # create generation time : gamma distribution, with mean 2.6 time units and standard deviation 1 time unit> GT.flu <− generation.time("gamma", c(2.6,1))> # loads example dataset> data(Germany.1918)> res.R <− estimate.R(Germany.1918, GT=GT.flu, methods=c("EG","ML","SB","TD"))# applies methods EG, ML, SB, TD to the dataset> plot(res.R) # diplays results> plotfit(res.R) # displays fit to the epidemic curve# sensitivity analysis according to choice of time window for exponential growth> sensitivity.analysis(Germany.1918, GT.flu, begin=1:15, end=16:30, est.method="EG", sa.type="time")> # sensitivity analysis according to generation time> sensitivity.analysis(Germany.1918, GT.type="gamma", GT.mean=seq(1,5,1), GT.sd.range=1, begin=1, end=27, est.method="EG", sa.type="GT")

## Abbreviations

AR: Attack rate; EG: Exponential growth; ML: Maximum likelihood; TD: Time dependent; SB: Sequential bayesian.

## Competing interests

The authors declare that they have no competing interests.

## Authors’ contributions

TO coded the software, wrote the manuscript. RH researched bibliography. PYB conceived the study, wrote the manuscript. All authors read and approved the final manuscript.

## Pre-publication history

The pre-publication history for this paper can be accessed here:

http://www.biomedcentral.com/1472-6947/12/147/prepub

## Supplementary Material

Additional file 1: Supplementary material S1 Imputation method for missing incidence values in the ML method. Click here for file
