# Supplementary material for: The R0 package: a toolbox to estimate reproduction numbers for epidemic outbreaks
Source: BMC Med Inform Decis Mak. 2012 Dec 18;12:147. doi: 10.1186/1472-6947-12-147 (PMC3582628; doi:10.1186/1472-6947-12-147)
Supplement: Additional file 1: Supplementary material S1 — Imputation method for missing incidence values in the ML method. [file 1472-6947-12-147-S1.docx]

Supplementary Material S1

Imputation method for missing incidence values in the ML method

We describe a framework that allows for correcting the bias in the reproduction number estimate occurring in method ML when the epidemic curve is not observed from the first case on.

Let $w=\left[ w_{0}, w_{1}, \ldots, w_{K} \right]$ denote the generation time distribution for the outbreak with a series of observed cases $N=[ N_{1}, N_{2}, \ldots, N_{T} ]$ (where $K\leq T$).

$I=[{EN}_{-K+1}, \ldots, {EN}_{-1} ,N_{0}, N_{1}, N_{2},\ldots, N_{K}, \ldots, N_{T} ]$ denote the outbreak augmented with expected incidence counts over the previous $K$ time units.

Equation [1] yields the observed number of cases at time *t*, for $t>K$ :

|  | $E\left( N_{t} \right)= R \left( \sum_{t-K\leq i<0} {EN}_{i}w_{t-i}+\sum_{0\leq i\leq t} N_{i}w_{t-i} \right)$ |  |
| --- | --- | --- |

We assume that the epidemic is in exponential growth, and model EN as a geometric progression according to :

$${EN}=e^{\beta}.[ \rho^{K-1}, \ldots, \rho,1]$$

where $\beta=\log\left( {EN}_{-1} \right)$ and$\rho= \frac{e^{\alpha}}{1+e^{\alpha}}$ .

The two parameters $\left\{ \alpha, \beta\right\}$ are estimated by minimizing

|  | $\sum_{t=1}^{K-1} \left( I_{t}-E\left( I_{t} \right) \right)^{2}$ |  |
| --- | --- | --- |

To illustrate the process of data augmentation we implemented, we deliberately erased the first 15 records of incidence from our example dataset.

> censored.Germany.1918<-Germany.1918[26:length(Germany.1918)]

Running the normal estimation leads to an estimate R=1.40 [ 1.30 ; 1.50 ]. With correction for the unobserved cases, the estimate is R=1.16 [ 1.11 ; 1.21 ], closer to the values obtained with the complete dataset (R = 1.21 [ 1.16 ; 1.27 ]).
